# Exploring the Lipids Involved in the Formation of Characteristic Lactones in Japanese Black Cattle

**DOI:** 10.3390/metabo11040203

**Published:** 2021-03-29

**Authors:** Shuji Ueda, Ryo Sasaki, Rio Nakabayashi, Minoru Yamanoue, Yasuhito Sirai, Eiji Iwamoto

**Affiliations:** 1 Department of Agrobioscience, Graduate School of Agricultural Science, Kobe University, Kobe 657-8501, Hyogo, Japan; yamanoue@kobe-u.ac.jp (M.Y.); shirai@kobe-u.ac.jp (Y.S.); 2 Food Oil and Fat Research Laboratory, Miyoshi Oil & Fat Co., Ltd., Tokyo 124-8510, Japan; sasakir@miyoshi-yushi.co.jp (R.S.); NAKABAYASHIR@miyoshi-yushi.co.jp (R.N.); 3 Hokubu Agricultural Technology Institute Hyogo Prefectural Technology Center for Agriculture, Forestry, and Fisheries, Asago 669-5254, Hyogo, Japan; eiji_iwamoto@pref.hyogo.lg.jp

**Keywords:** Japanese black cattle, lipidomics, gas chromatography-olfactometry, wagyu beef aroma, triacylglyceride

## Abstract

The meat from Japanese Black cattle (Japanese Wagyu) is finely marbled and exhibits a rich and sweet aroma known as Wagyu beef aroma. To clarify the key metabolites involved in the aroma, we analyzed the correlation between lactone and lipid composition in Japanese Black cattle. Using gas chromatography-olfactometry, we identified 39 characteristic odorants of the intermuscular fat. Seven characteristic lactones considered to be involved in Wagyu beef aroma were quantified and compared in the marbled area and intermuscular fat using a stable isotope dilution assay. Among them, γ-hexalactone was the only lactone whose level was significantly higher in the marbled area. To explore the lipid species involved in lactone formation, we analyzed samples with different aroma characteristics. Liquid chromatography-mass spectrometry revealed eight lipid classes and showed significant differences in triacylglycerides (TAGs). To determine the molecular species of TAGs, we performed high-performance liquid chromatography analysis and identified 14 TAG species. However, these analyses showed that seven lactones had a low correlation with the TAGs. However, γ-hexalactone showed a positive correlation with linoleic acid. This study suggests that lipid composition affects the characteristic lactone profile involved in the Wagyu beef aroma.

## 1. Introduction

Japanese Black cattle, also known as Japanese Wagyu, are used to produce one of the world’s most renowned types of beef [1]. Its defining characteristics are the excellent marbling of crossed fat in muscle tissues and its rich and sweet aroma (the so-called Wagyu beef aroma). Matsuishi et al. confirmed that lactones of cyclic esters produced during cooking contributed to the Wagyu beef aroma [2]. There are two types of lactones produced during beef cooking, namely, γ-lactone and δ-lactone, and they have different heterocyclic carbon atoms. The quality of the sweet odor changes with the length of the carbon chain bonded to the heterocycle [3].

Holstein cattle are bred as dairy cattle, but male cattle castrated before weaning are used for meat production. Holstein cattle, which are mostly characterized by lean meat, are often used experimentally in comparisons with Japanese Black cattle, as their proportions of intramuscular fat [4], which is responsible for marbling in the muscles, differ significantly. Japanese Black cattle have a considerably higher marbling rate than Holstein cattle and are rich in monounsaturated fatty acids (MUFAs) [5]. As the characteristic lipid metabolism of Japanese Black cattle has a significant influence on meat quality (tenderness, flavor, and juiciness) [6,7], there are several studies on genes which are crucial for the marbling trait [8,9,10,11]. In addition, studies have attempted to analyze the aroma of Wagyu beef [12,13,14]. The aroma analysis of odorants, which are volatile compounds with different boiling points, requires the following two operations: a selective collection of the odorants generated from the beef and precise mass spectrometry to determine their molecular weights.

Previously, odorants associated with Wagyu beef aroma were collected using solvent-assisted flavor evaporation (SAFE) at ultralow temperatures to prevent volatilization [15]. In such aroma analyses, a combination of gas chromatography-olfactometry (GC-O) technology and GC-tandem quadrupole mass spectrometry was used to detect odorants that were generated from the cooked fat tissues of Japanese Black cattle. This GC-O technology has been used to evaluate the aromas of foods; moreover, it is useful when evaluating the characteristics of volatile components [16]. Previous studies focused on metabolites (sugars, organic acids, and amino acids) [17] that are correlated with Wagyu beef aroma [11], but not the effects of lipid composition. Lipid composition is the most characteristic trait of Japanese Black cattle, and the effects that MUFAs may have on beef aromas represent an interesting topic in current livestock research [18,19].

In this study, we aim to investigate the relationship between lipid composition and Wagyu beef aroma using intermuscular fat and marbled area of steak to clarify the key metabolites contributing to Wagyu beef aroma development. The lipid classes and major triacylglyceride (TAG) species in Japanese Black cattle were identified by liquid chromatography-mass spectrometry (LC-MS) and high-performance liquid chromatography (HPLC). Furthermore, by comparing the lipid analysis data with the quantified lactones using a stable isotope dilution assay (SIDA), the lipid species with a positive correlation with Wagyu beef aroma were examined.

## 2. Results and Discussion

### 2.1. GC-O Analysis of Intermuscular Fat Aroma

In the gas chromatography-mass spectrometry (GC-MS) analysis, the aroma components from Japanese Black cattle ribeye steak (musculus longissimus) formed more than 1000 peaks after cooking, making it difficult to identify the odorants that contributed to Wagyu beef aroma; see Figure S1. Consequently, the ribeye steak was divided into two parts, namely, intermuscular fat around the steak and marbled beef (called marbled area in this study).

In this study, we hypothesized that lipids are involved in the formation of Wagyu beef aroma. The aroma characteristics of the intermuscular fat were analyzed using GC-O analysis. The focus was on the 39 odorants previously detected in the marbled area using the GC-O analysis [11]. Similar to previous data, the odorants generated when the boiled intermuscular fat of Japanese Black cattle of different pedigrees (Type A and Type B) and Holstein cattle were compared.

Of the 39 odorants, 36 were detected in the intermuscular fat of Type A and Type B Japanese Black cattle and Holstein cattle. These odorants were confirmed by comparing their odor quality and retention index (RI) on the Agilent DB-Wax capillary column Table 1. When the flavor dilution (FD) factors were compared, 11 odorants (**3**, **4**, **6**, **17**, **18**, **23**, **27**, **31**, **34**, **36**, and **38**) were detected at higher FD values in Japanese Black cattle (both Type A and Type B) than in Holstein cattle. Alternatively, only hexanal (**2**), whose odor quality resembles freshly cut grass [20], was detected at a higher FD value in Holstein cattle than in Japanese Black cattle. The characteristics of the odorants detected with a high FD value in Japanese Black cattle were as follows: δ-decalactone (**31**) has a sweet milk aroma, and it is abundant in dairy products; 2-acetyl-1-pyrroline (**6**) and 2-acetyl-1-thiazoline (**18**) are nitrogenous compounds produced by the Maillard reaction, and they have a popcorn-like roasted odor [21]; indole (**36**) and 3-methylindole (**38**) are heterocyclic amines, and they mostly have a unique animal odor, with a slight floral odor [22]. Our GC-O analysis revealed that lactone-based odorants, such as γ-octalactone (**23**), γ-nonalactone (**27**), and δ-decalactone (**31**), and indole-based odorants, such as indole (**36**) and 3-methylindole (**38**), may contribute to the characteristic sweet aroma generated from the fat tissue (beef tallow) of Japanese Black cattle.

In all tissues assessed of Type A and B Japanese Black and Holstein cattle, eight odorants (**5**, **8**, **9**, **11**, **12**, **17**, **21**, and **26**) were detected with FD values (≥6). These odorants were also detected with a high FD value (≥6) in marbled areas in previous studies. Of these odorants, 3-methyl-3-furanthiol (5) is a thiol compound produced by the Maillard reaction [23]. The other odorants (**8**, **9**, **11**, **12**, **17**, **21**, and **26**) are aliphatic aldehydes that are produced by the degradation of *γ*-hydroxycarboxylic acids derived from oxidative fatty acids [24]. These odorants, with a high FD values in both intermuscular fat and marbled area, are considered a common basis for the aroma of boiled beef.

**Figure 1:**
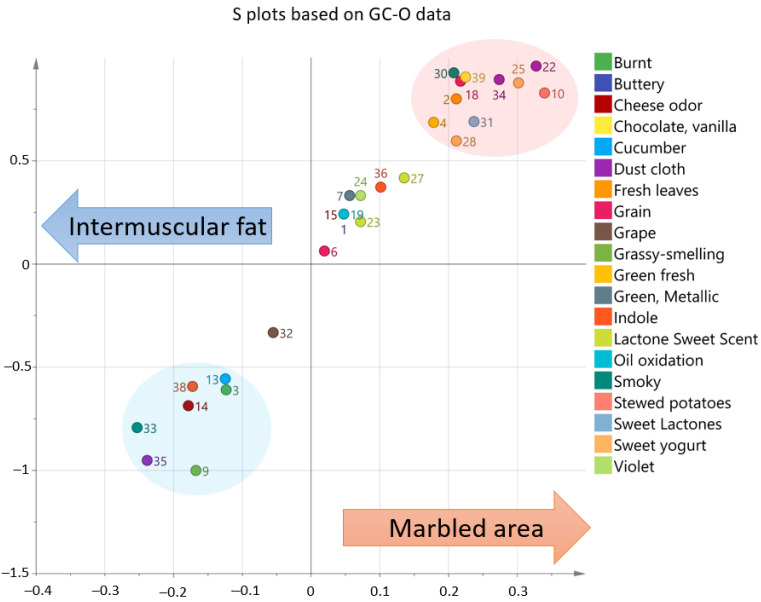
S-shaped plot for the orthogonal part least squares discrimination analysis (OPLS-DA). Fat tissues and muscle tissues from the musculus longissimus of Japanese Black cattle Type A and Type B, and Holstein cattle were used for the analysis. The multivariate analysis showed characteristic odorants in the intermuscular fat and marbled area (R2X = 0.474, Scaling, Par). The OPLS-DA model was calculated based on the data presented in Table 1 and previous study data [11]. The score plots of the OPLS-DA model were R2 (cum) = 1.00 and Q2 (cum) = 0.997. The plot number indicates the number of compounds in the corresponding odorants Table 1.

The characteristic odorants of intermuscular fat and marbled area are displayed as visual plots using the multivariate analysis of their FD values in Figure 1. The left side of the S plot shows the prominent odorants (**3**, **9**, **13**, **14**, **33**, **35**, and **38**) of the intermuscular fat. Among them, 3-methyl-2-butene-1-thiol (**3**) is a sulfur compound that causes the aging flavor of beer [25], whereas 4-vinylphenol (**33**) and 9-decenoic acid (**35**) have unique flavors. These unique odorants (**3**, **33**, and **35**), which have high FD values in intermuscular fat, may contribute to the distinctive complex aroma generated from fat tissues [26]. In contrast, the right side shows the prominent odorants of marbled area (**2**, **4**, **10**, **18**, **22**, **25**, **28**, **30**, **31**, **34**, and **39**). Methional (**10**) is a degradation compound of methionine, generating a stewed potato odor [27]. Maltol (**25**) is a caramel-like odorant produced by the Maillard reaction; it is also known to have an e-cigarette fragrance [28]. Hexanoic acid (**22**) and decanoic acid (**34**) are medium-chain fatty acids with a dust cloth odor [29]. These odorants (**10**, **22**, **25**, and **34**) are inferred from the odor quality associated with the beef flavor specific to the muscle tissue.

### 2.2. Quantification of Odorants Using the Stable Isotope Dilution Assay (SIDA)

**Figure 2:**
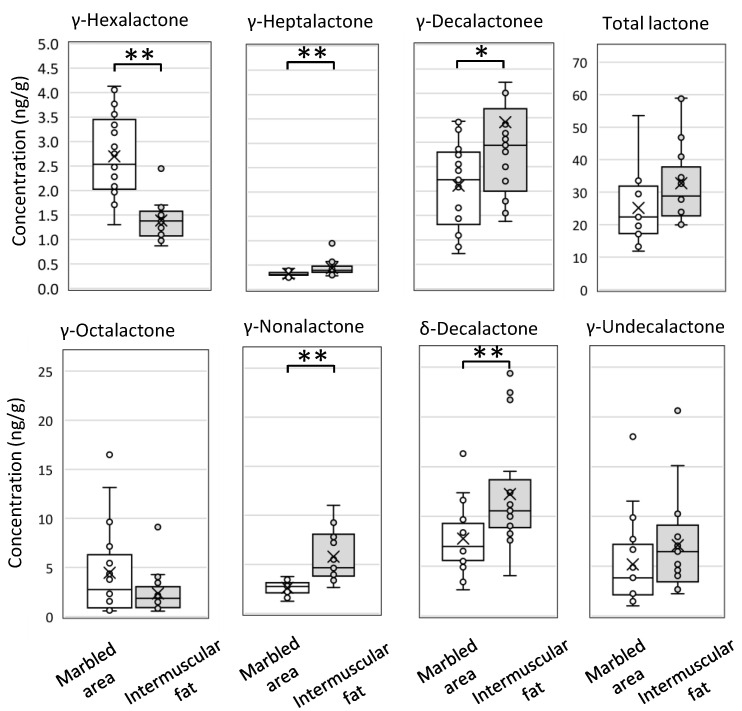
Comparative analysis of the characteristic odorants identified in the intermuscular fat and marbled area of Japanese Black cattle. The lactones were measured after boiling using the stable isotope dilution assay (SIDA). The box plot is an exclusive median and shows all plots, including outliers. The cross marks indicate the mean values. The samples analyzed were the intermuscular fat and marbled area from Japanese Black cattle (17 cattle for each tissue, 10 Type A and 7 Type B). Type A Japanese Black cattle are a typical pedigree (non-Tajima), and Type B is a closed breeding pedigree (Tajima). The total lactone is the sum of the γ-hexalactone, γ-heptalactone, γ-octalactone, γ-nonalactone, γ-decalactone, δ-decalactone, and γ-undecalactone. Significant differences are indicated as follows: ** *p* < 0.01, * *p* < 0.05.

In a previous study, the lactones related to beef aroma were studied using solvent-extracted lipids [14]. In this study, we compared intermuscular fat and marbled area to examine the lactone characteristic of Wagyu beef aroma generated from the edible part. The box plots in Figure 2 show the seven lactones measured using the SIDA. Although γ-hexalactone (**16**), γ-heptalactone (**20**), γ-decalactone (**29**), and γ-undecalactone (**ND**) were not or were slightly detected in the intermuscular fat using the GC-O analysis Table 1, these lactones could be quantified using the SIDA. γ-Heptalactone (**20**), γ-decalactone (**29**), γ-nonalactone, δ-decalactone, and γ-undecalactone were detected at higher levels in the intermuscular fat than in the marbled area. Whereas, γ-hexalactone (**16**) and γ-octalactone (**23**) presented higher levels in the marbled area than in the intermuscular fat. The level of γ-hexalactone was the highest in the edible marbled area (*p* < 0.001).

Lactones are sweet odorants that are produced from hydroxy fatty acids [30]. Their volatility depends on the number of carbon atoms. γ-Hexalactone is the most volatile lactone (boiling point 220 °C) and exhibits a sweet fruity and nutty aroma [31]. Previous studies have shown that γ-hexalactone correlates with the strength of Wagyu beef aroma and proposed that γ-hexalactone can be an indicator of Wagyu beef aroma [11]. The difference in lactone profiles determined using the SIDA is considered to reflect the quality of Wagyu beef aroma between the fat and muscle tissue. As the marbled area is the edible part, we focused on that in the next comprehensive analysis of lipid composition.

### 2.3. Lipidomic Analysis of Japanese Black Cattle Meat by Liquid Chromatography-Mass Spectrometry (LC-MS)

To comprehensively detect multiple lipid classes in the marbled area of Japanese Black cattle meat, we used LC-MS analysis. For this analysis, we selected loin (musculus longissimus) and adductor magnus (round) as the negative control from the same Japanese Black cattle to match the genetic and physiological conditions. The musculus longissimus has more marbling than the adductor magnus, and it is highly valued for its flavor [32]. We also added samples of different meat quality grades (A3 and A5 rank) to the comparison. A5 is the highest quality grade, and A3 is the middle grade with a moderate marbling rate [33].

**Figure 3:**
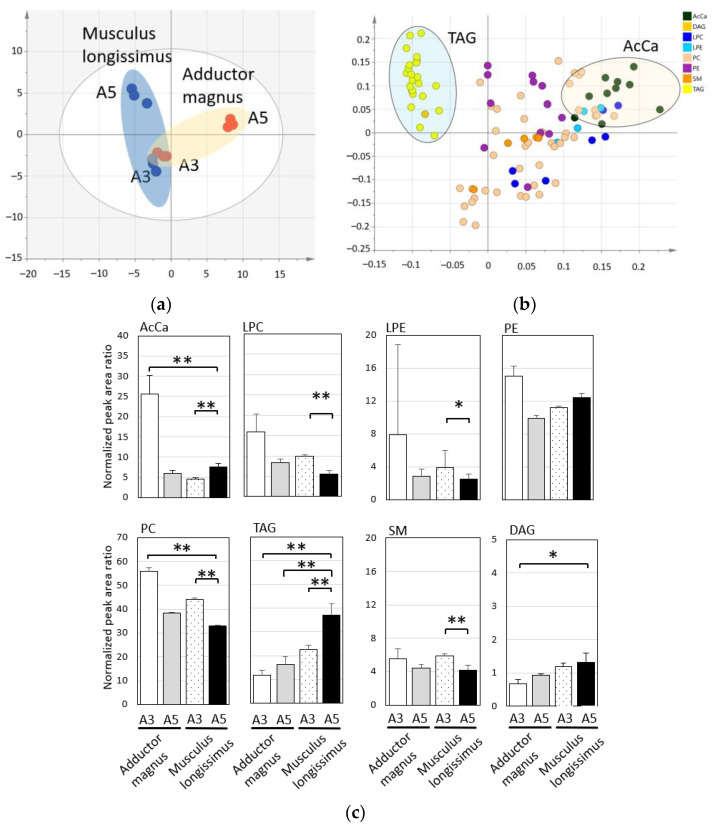
Lipid characteristics of Japanese Black cattle muscle tissues. The samples used for analysis were adductor magnus and musculus longissimus from Japanese Black cattle (three cattle in each sample). (**a**) Principal component analysis (PCA) score plot visualizing the relationship between the adductor magnus and musculus longissimus samples using lipid molecular species (scaling, Par). (**b**) Loading plot showing the relationship between the lipid molecular species and the samples. The PCA model was used for analysis with the fitting parameters (R2X (1) = 0.535; R2X (2) = 0.238). (**c**) The total amount of each lipid class was calculated from the peak area of the lipid molecular species identified by LC-MS. The graph shows the mean and standard deviations of the total lipid class. Significant differences are indicated as follows: ** *p* < 0.01, * *p* < 0.05. Abbreviations: acylcarnitine (AcCa), lysophosphatidylcholine (LPC), lysophosphatidylethanolamine (LPE), phosphatidylcholine (PC), phosphatidylethanolamine (PE), sphingomyelin (SM), diacylglyceride (DAG), triacylglyceride (TAG).

After normalization and removal of the nonspecific peaks, our LC-MS analytical conditions revealed 108 lipids in the chloroform/methanol fraction. The mean value of the coefficient of variation for the internal standards was 8.1%, and good reproducibility was confirmed. The lipid species identified by LC-MS were classified into eight classes; see Figure S2. The molecular species in the lipid class included the following: acylcarnitine (AcCa), 10 species; lysophosphatidylcholine (LPC), 9 species; lysophosphatidylethanolamine (LPE), 4 species; phosphatidylcholine (PC), 44 species; phosphatidylethanolamine (PE), 12 species; sphingomyelin (SM), 5 species; diacylglyceride (DAG), 1 species; and triacylglyceride (TAGs), 23 species; see Figure 3. However, it was difficult to accurately separate the TAG molecular species because their three fatty acids were bound to the glycerol skeleton in various combinations.

AcCa and PC were significantly higher in the adductor magnus with less marbling than in the samples. The tendency for the PC values to be higher in the adductor magnus was consistent with that observed in the analysis of phospholipids in New Zealand beef [34]. AcCa consists of carnitine and fatty acids and is involved in the β-oxidation of lipids. AcCa is abundant in the mitochondria, particularly in lean muscle [35]. In contrast, TAG was significantly higher in the musculus longissimus, depending on the meat quality grade (level of marbling). TAG is the major lipid stored in the lipid droplets of adipocytes. Lipid droplets have a single membrane structure of phospholipids surrounding the hydrophobic TAG and cholesterol esters [36]. This difference in lipid composition, consisting of lipid droplets, may be reflected in the LC-MS results. It is known that the A5 musculus longissimus has a strong Wagyu beef aroma [7,37]. Next, we focused on the TAG, which is the most abundant in A5 musculus longissimus, and measured its molecular species composition.

### 2.4. Quantitative Analysis of the Fatty Acid and TAG Molecular Species Compositions and Odorants

To explore the lipids involved in the Wagyu beef aroma, we analyzed the correlation between TAG composition and lactones generated from the marbled area. First, the fatty acid composition of the TAG fraction prepared from the musculus longissimus of Japanese Black cattle was analyzed by gas chromatography. Their fatty acid compositions are shown in Table 2. MUFAs accounted for 54.4% of the total fatty acid content. Oleic acid (C18:1) 48.8%, palmitoleic acid (C16:1) 4.5%, and myristoleic acid (C14:1) 1.1% were identified as the major MUFAs. Saturated fatty acids (SFAs) accounted for the remaining 38.3%, and polyunsaturated fatty acids (PUFAs) accounted for 2.9%. Of the SFAs, palmitic acid (C16:0) 24.9%, stearic acid (C18:0) 9.7%, and myristic acid (C14:0) 2.6% were detected. Of the PUFAs, linoleic acid (C18:2) 2.6% was higher than linolenic acid (C18:3) 0.3%. The odd-carbon fatty acids, pentadecanoic acid (C15:0) 0.3%, and margaric acid (C17:0) 0.8% were slightly detected.

Next, we used high-performance liquid chromatography (HPLC) to analyze TAG molecular species; this approach is conventionally used to separate TAG molecules [38]. After normalization, the HPLC analytical conditions revealed 14 peaks in the TAG fraction; see Figure S2. The details of the TAGs are shown in Table 2. The major TAGs were POO, 29.9%; POP, 9.8%; PPoO, 8.2%; POS, 7.9%; SOO, 7.3%; OOO, 7.2%; MOP, 4.9% (O, oleic acid; P, palmitic acid; S, stearic acid; Po, palmitoleic acid; and L, linoleic acid). Seven TAGs accounted for 75.2% of the total TAGs. The total peak area for the unknown TAGs was 10.2%. To clarify the TAG contributing to Wagyu beef aroma, we quantified the above seven lactones using the SIDA. The lactone levels produced from musculus longissimus during boiling are shown in Table 2.

### 2.5. Correlation between the Composition of Lipids and Odorants Related to Wagyu Beef Aroma

Next, we examined the correlations among TAGs, fatty acid composition, and the seven lactones. As expected, the TAG molecular species and fatty acid compositions showed a high correlation; see Table 3. Of the fatty acids, myristic acid, stearic acid, linoleic acid, palmitoleic acid, and margaric acid presented low TAG composition ratios. These fatty acids showed a high correlation with specific TAGs as follows: myristic acid (MOP: *r* = 0.98), stearic acid (SOS: *r* = 0.97), POS (*r* = 0.92), linoleic acid (PLO: *r* = 0.93), palmitoleic acid (PPoO: *r* = 0.92), and margaric acid (POMa: *r* = 0.88). The negative correlations were as follows: stearic acid (PPoO: *r* = −0.94), palmitoleic acid (POS: *r* = −0.88), POO (*r* = −0.87), (SOS: *r* = −0.84), and myristoleic acid (POO: *r* = −0.84). Unknown TAG was highly correlated with myristoleic acid (*r* = 0.90) and palmitoleic acid (*r* = 0.89) of MUFAs. This MUFA correlation suggests the presence of TAG containing MUFA at the Unknown peak, but we were not able to identify them. In contrast, TAG and lactones unexpectedly showed a low correlation. γ-Decalactone showed a positive correlation with PPoO (*r* = 0.42) and unknown TAG (*r* = 0.41), and γ-hexalactone showed a positive correlation with PLO (*r* = 0.40). γ-Hexalactone showed a negative correlation with MOP (*r* = −0.50), γ-decalactone showed a negative correlation with SOO (*r* = −0.43) and SOS (*r* = −0.43), and γ-heptalactone showed a negative correlation with PPoO (*r* = −0.42).

In the correlation between fatty acid composition and lactones, several fatty acids were correlated. γ-Hexalactone was positively correlated with linoleic acid (*r* = 0.54) and negatively correlated with myristic acid (*r* = −0.53). Among other lactones, γ-decalactone was correlated with myristoleic acid (*r* = 0.54) and linolenic acid (*r* = −0.49). The heatmap overview showed a similar tendency for low molecular weight γ-hexalactone (six carbon atoms; C-6) and γ-heptalactone (C-7). PUFAs linoleic acid and linolenic acid were correlated with γ-hexalactone and γ-heptalactone. The cyclization of hydroxy fatty acids generally causes the formation of γ-lactones [39]. γ-Nonalactone (C-8) and γ-decalactone (C-10) are formed via hydroxy fatty acids during fermentation from MUFAs, such as oleic acid and palmitoleic acid [30]. Unlike other γ- lactones, the details of the molecular mechanism of γ-hexalactone formation while cooking beef are unknown. From the results of the correlations between fatty acids and γ-hexalactone, it is speculated that PUFAs are involved in the formation of low molecular weight γ-lactones. In addition, as γ-hexalactone is produced in a marbled area, it is suggested that metabolites (organic acids, amino acids, and sugars) derived from muscle tissue are involved [17]. Metabolites (glutamine, decanoic acid, sedoheptulose 7-phosphate, creatinine, and xanthine) that are positively correlated with γ-hexalactone in muscle tissue may be candidates involved in the formation of low molecular weight γ-lactones [11]. The production of hydroxy fatty acids may also contribute to lipase activity, resulting in the production of free fatty acids in the muscle tissue of the marble area.

In this study, we did not find a strong correlation between the TAG molecular species and the seven lactones. As more combinations can be inferred from the fatty acid compositions, the presence of many unidentified TAG molecular species is expected. However, γ-hexalactone showed a positive correlation with linoleic acid. This study suggests that lipid composition affects the characteristic lactone profile involved in Wagyu beef aroma.

## 3. Materials and Methods

### 3.1. Sample Collection

**Figure 4:**
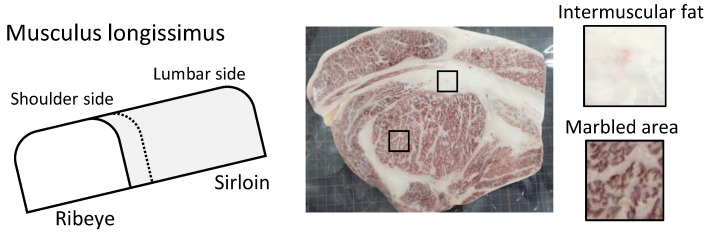
Marbled area and intermuscular fat in Japanese Black cattle. The dotted line in the schematic diagram shows a cross-sectional position of the right photograph of musculus longissimus. The photograph shows the ribeye steak used in this study. Arrows indicate intramuscular fat in the marbled area.

We purchased the muscle blocks commercially from meat wholesalers. The blocks were then sliced into steaks and individually packaged. These samples were then stored in a freezer at −30°C (Table 4 and Figure 4).

### 3.2. GC-O Analysis of the Odorant Concentrations in Boiled Beef

As in a previous report [11], the aroma characteristics of Wagyu beef were compared with those of the intermuscular fat from Type A and Type B Japanese Black cattle and Holstein cattle. A total of 50 g of fat tissue (intermuscular fat around the steak) was boiled in 500 mL of distilled water for 30 s. The fat tissues were cooled with ice, ground with a mixer with 500 mL water containing the fat eluted by boiling, added to 500 mL dichloromethane, and extracted for 16 h at 25 °C with stirring. Many nonvolatile compounds, such as the fats and oils derived from the fat tissues, were removed using an SAFE apparatus at an ultralow temperature (−196 °C). After drying with anhydrous sodium sulfate, the sample was concentrated using a Kuderna-Danish evaporative concentrator [11].

The GC-O analysis was performed under the same conditions to compare the data with those of a previous analysis that used the marbled area [11]. For the GC-O analysis, we used CharmAnalysis (DATU, Geneva, NY, USA) with an Agilent 6890 gas chromatograph (Agilent Technologies, Santa Clara, CA, USA) equipped with a DB-WAX capillary column (length, 15 m; inner diameter, 0.32 mm; film thickness, 0.25 mm; Agilent Technologies) [15]. The odor extract was diluted stepwise (four-fold) with dichloromethane. Compounds were identified by comparing their odor quality, RI, and mass spectrum with those of authentic compounds on a DB-WAX column.

### 3.3. Quantification of Lactone

The concentration of the odorants was measured using the SIDA with a liquid extraction method in which stable isotopes were added to dichloromethane as an internal standard. Stable isotopes of γ-nonalactone, γ-octalactone, γ-decalactone, γ-undecalactone, γ-hexalactone, γ-heptalactone, and δ-decalactone were purchased from AromaLAB (Martinsried, Germany). Quantitation was performed using a GC-tandem quadrupole mass spectrometer (Agilent 7000C TripleQuad GC-MS system; Agilent Technologies) equipped with a DB-WAX capillary column (length, 30 m; inner diameter. 0.25 mm; film thickness, 0.25 mm; Agilent Technologies). The oven temperature was programmed to hold at 35 °C for 5 min, and then increased from 35 °C to 217 °C at a rate of 4 °C/min. The system was operated in the multiple reaction monitoring (MRM) mode. Two microliters of the concentrate were then injected into the instrument with the inlet temperature set at 250 °C in the spitless mode.

### 3.4. LC-MS Analysis

One gram of adductor magnus or musculus longissimus was frozen in liquid nitrogen, and then milled with a multibead shocker μT-48 (Token, Chiba, Japan). The powdered samples (20 mg) were homogenized with 500 μL methanol using an ultrasonic cleaner for 10 min and stirred at 2500 rpm for 5 min in a shaking incubator. Chloroform (500 μL) was then added, and they were shaken at 2500 rpm for 5 min in a shaking incubator and centrifuged at 9100× *g* for 5 min to obtain a chloroform/methanol fraction. A sample was prepared by adding an internal standard of phosphatidic acid (16:0 D30/18:1) to the supernatant to a final concentration of 0.2 μg/mL. The internal standard was purchased from Avnati Polar Lipids (Alabaster, AL, USA). High-resolution Fourier transform mass spectrometry (LC-MS) analysis consisted of ultrafast liquid chromatography (UFLC XR; Shimadzu, Kyoto, Japan) with an L-column2 ODS metal-free column (inner diameter, 2 mm; length, 50 mm; particle size, 3 μm; Chemicals Evaluation and Research Institute, Tokyo, Japan) and LTQ-Orbitrap XL (Thermo Fisher Scientific K. K., Tokyo, Japan). The separation buffer for the UFLC consisted of solvent A (1 mM ammonium formate solution), solvent B (ammonium formate/isopropanol), and solvent C (acetonitrile). For the UFLC, the injection volume was 3 μL, sampler temperature was 4 °C, column temperature was 40 °C, and flow rate was 0.3 mL/min. Mass spectrometry was performed using the thermal electrospray ionization method in the data-dependent Top N3 scan mode at a resolution of 30,000 from 200 to 1600 *m*/*z*.

For peak selection, the estimations for the lipid species and alignments between the samples were analyzed using the lipid identification software Lipid Search (Mitsui Information, Tokyo, Japan). The search options were set as follows: precursor tolerance, 5.0 ppm; product tolerance, 0.5 Da; merge range, 2.0; and min peak width, 0.0. The quantitation option was set as follows: MZ tolerance, −5.0 to +5.0 ppm; RT range, −0.5 to +0.5 min. The estimated lipids were confirmed based on the retention time and product ions. In this study, only lipids satisfying the conditions (peak intensity ≥5000, coefficient of variation ≤0.15, and mean signal-to-noise ratio ≥3) were selected. The area of the detected peak in each sample was corrected using the peak area value of the internal standard [41].

### 3.5. Analysis of Fatty Acids and TAG Composition

Total lipids were extracted from ground beef (10 g) with t-butyl methylether/methanol (2:1) according to a previously described method [42]. TAG fractions were collected using solid phase extraction with an InertSep SI column (GL Sciences, Tokyo, Japan) according to the manufacturer’s instructions. To analyze the fatty acid composition of the TAG samples, they were first dissolved in n-hexane and methyl esterified with potassium hydroxide-methanol solution [43]. The methylated sample (1 μL) was used for gas chromatography (GC-2010 Plus; Shimadzu, Kyoto, Japan) with a TC-70 capillary column (length, 60 m; inner diameter, 0.25 mm; film thickness, 0.25 μm; GL Sciences). The initial temperature was set at 150 °C, increased at 5 °C/min to 235 °C, and then held at that temperature for 8 min. The oven temperature was maintained at 150 °C for 30 min and was then increased to 250 °C at a rate of 10 °C/min and held isothermally for 13 min.

For the TAG analysis, the dissolved samples in isopropyl alcohol were subjected to HPLC using Agilent Technologies 1260 Infinity equipped with a refractive index detector and a Poroshell 120 EC-C18 LC column (three columns in a series, 3.0 mm × 50 mm, 3.0 mm × 50 mm, 3.0 mm × 100 mm; 2.7-Micron; Agilent Technologies). A mixture of acetonitrile and 2-propanol (4:6, *v*/*v*) was used. The flow rate was 0.2 mL/min, and the column temperature was maintained at 20 °C. The quantification of individual TAGs was performed by evaluating the corresponding relative percentage according to the normalization area procedure.

### 3.6. Statistical Analysis

Statistical significance was determined using a *p*-value (Student’s *t*-test and Bonferroni test) with Excel 2019 software (Microsoft Japan, Tokyo, Japan). The correlation coefficient and uncorrelated test variables between lactones and lipids were recalculated using JMP12 (SAS Institute Japan, Tokyo, Japan). Multivariate data analysis of OPLS-DA, and PCA was performed using SIMCA14 software (Inforcom, Tokyo, Japan).

## 4. Conclusions

In this study, we compared and characterized 39 odorants that were detected using the GC-O analysis from the intermuscular fat and marbled area from Type A and Type B Japanese Black cattle and Holstein cattle. The GC-O analysis revealed various odorants that contribute to the flavor of cooked beef generated from the fat and muscle tissue. The qualitative analysis using SIDA revealed seven odorants that contributed to Wagyu beef aroma, and γ-hexalactone was identified as a characteristic odorant generated from the marbled area. The lipid composition was investigated, as it was expected to be involved in the formation of γ-hexalactone. The LC-MS analysis revealed 108 lipids belonging to eight lipid classes in adductor magnus and musculus longissimus from Japanese Black cattle. The semiquantitative analysis using HPLC revealed 14 TAGs contained in the marbled area. Contrary to our hypothesis, the correlation of the γ-hexalactone content with the major TAG species was small but positive (*r* = 0.55) for linoleic acid and negative (*r* = −0.53) for myristic acid.

## Figures and Tables

**Table 1:** Detection of 39 odorants derived from intermuscular fat by gas chromatography-olfactometry (GC-O).^a^ RI is the retention index of the DB-WAX column used in gas chromatography-mass spectrometry (GC-MS).^b^ Flavor dilution (FD) factors determined under the supervision of scent experts.^c^ Fragrance detected at the sniffing port during GC-O.^d^ ND indicates that it was not detected in either Japanese Black cattle or Holstein cattle.

Intermuscular Fat			Odor Quality ^c^	FD Factor ^b^ (Log_4_)			Relative Value (Type B/Holstein)
				Japanese Black Cattle			
No.	RI ^a^	Conpound		Type A	Type B	Holstein	
1	983	2,3-Butanedione	Buttery	3	3	3	1.0
2	1105	Hexanal	Fresh leaves	2	2	3	0.7
3	1123	3-Methyl-2-butene-1-thiol	Burnt	1	2	0	-
4	1291	Octanal	Green fresh	1	2	0	-
5	1299	2-Methyl-3-furanthiol	Nuts	7	7	7	1.0
6	1331	2-Acetyl-1-pyrroline	Grain	0.3	5	0	-
7	1371	1,5-Octadien-3-one	Green, Metallic	4	5	4	1.3
8	1423	Nonanal	Oil oxidation	7	7	7	1.0
9	1438	(E)-2-Octenal	Grassy-smelling	6	6	6	1.0
10	1441	Methional	Stewed potatoes	0	5	0	-
11	1501	Decanal	Green fresh	7	7	7	1.0
12	1530	(E)-2-Nonenal	Oil oxidation	7	7	7	1.0
13	1577	(2E,6Z)-Nona-2,6-dienal	Cucumber	4	5	4	1.3
14	1623	Butyric acid	Cheese odor	4	4	4	1.0
15	1669	Isovaleric acid	Cheese odor	1	1	1	1.0
16	1684	γ-Hexalactone	Sweet, milk.	0	0	0	ND ^d^
17	1690	(2E,4E)-2,4-Nonadienal	Oil oxidation	7	7	6	1.2
18	1731	2-Acetyl-1-thiazoline	Grain	2	1	0.3	3.3
19	1757	2-Undecenal	Oil oxidation	2	4	2	2.0
20	1787	γ-Heptalactone	Sweet, milk.	0	0	0	ND
21	1800	(E,E)-2,4-Decadienal	Oil oxidation	7	7	7	1.0
22	1842	Hexanoic acid	Dust cloth	2	2	2	1.0
23	1890	γ-Octalactone	Lactone Sweet Scent	5	6	0.3	20.0
24	1928	β-Ionone	Violet	0.3	2	0.3	6.7
25	1941	Maltol	Sweet yogurt	0.3	0.3	0.3	1.0
26	1989	4,5-Epoxy-2(E)-decenal	Metal	7	7	7	1.0
27	2004	γ-Nonalactone	Lactone Sweet Scent	1	5	0.3	16.7
28	2021	Franeol	Sweet yogurt	0	5	0	-
29	2099	γ-Decalactone	Lactone Sweet Scent	0.3	0.3	0.3	1.0
30	2171	4-Vinyl guaiacol	Smoky	5	5	5	1.0
31	2185	δ-Decalactone	Sweet Lactones	3	5	0.3	16.7
32	2188	2-Aminoacetopheone	Grape	2	3	3	1.0
33	2256	4-Vinyl phenol	Smoky	3	5	4	1.3
34	2288	Decanoic acid	Dust cloth	2	2	1	2.0
35	2361	9-Decenoic acid	Dust cloth	2	3	2	1.5
36	2368	Indole	indole	4	3	0	-
37	2445	3-Methoxyphenol	Vanilla	0	0	0	ND
38	2459	3-Methylindole	Indole	4	3	0	-
39	2537	Vanillin	Chocolate, vanilla	4	5	5	1.0

**Table 2:** Summary data of the triacylglycerides (TAGs), fatty acids, and lactones identified in Japanese Black cattle. Musculus longissimus of Japanese Black cattle (total 20 cattle: 10 Type A and 10 Type B) were used in the analysis. The data are shown as mean and standard deviation of the quantification values. The three uppercase letters in the table on the left indicate the structure of TAG as a combination of ester-bonded fatty acids. These notations do not reflect the position of the glycerol skeleton of fatty acids. The TAG in parentheses indicates the TAG molecular species expected to be mixed slightly owing to similar retention times as follows: ^a^ POO (+ SLO), ^b^ POP (+ PLS), ^c^ PPoO (+ MOO), ^d^ MOP (+ PLP). ^e^ Unknown or other indicates the total peak area of the unidentified molecular species.

Triacylglyceride (%)		Muscle Tissue		
POO ^a^		29.9	±	1.7
POP ^b^		9.8	±	1.1
PPoO ^c^		8.2	±	1.0
POS		7.9	±	1.5
SOO		7.3	±	1.1
OOO		7.2	±	1.6
MOP ^d^		4.9	±	0.7
OOPo		4.0	±	1.2
PLO		3.1	±	0.6
PPP		2.9	±	0.4
SOS		1.7	±	0.5
PPS		1.3	±	0.3
POMa		1.3	±	0.2
SSS		0.1	±	0.1
Unknown ^e^		10.2	±	1.7
**Fatty Acid (%)**	**Symbol**	**Muscle Tissue**		
C18:1	O	48.8	±	2.2
C16:0	P	24.9	±	1.9
C18:0	S	9.7	±	1.4
C16:1	Po	4.5	±	0.9
C14:0	M	2.6	±	0.5
C18:2	L	2.6	±	0.6
C14:1	Mo	1.1	±	0.3
C17:0	Ma	0.8	±	0.2
C15:0	Pe	0.3	±	0.1
C18:3	Al	0.3	±	0.1
Other ^e^	-	4.4	±	0.5
**LACTONES (ng/g Beef)**		**Muscle Tissue**		
γ-hexalactone		2.6	±	0.9
γ-heptalactone		0.3	±	0.1
γ-decalactone		2.1	±	0.8
γ-octalactone		4.0	±	4.4
γ-nonalactone		2.5	±	0.7
δ-decalactone		8.5	±	3.8
γ-undecalactone		4.5	±	4.4

**Table 3:** Heatmap summarizing the relationships among triacylglycerides (TAGs), fatty acids, and odorants. The correlation coefficients among the TAG molecular species, their fatty acid compositions, and the quantitative values of seven lactones related to Wagyu beef aroma were examined. Red indicates a strong positive correlation and blue indicates a strong negative correlation. The symbols in the middle column are abbreviated fatty acids. ^a^ POO (+ SLO), ^b^ POP (+ PLS), ^c^ PPoO (+ MOO), ^d^ MOP (+ PLP). ^e^ Unknown or other indicates the total peak area for unidentified molecular species. The dotted frame shows the result of the significance test (*p* < 0.05). The thick black frame shows the result of the significance test (*p* < 0.0001). The color of the heatmap reflects the value of the correlation coefficient. Red color indicates positive correlation, and blue color indicates negative correlation.

Muscle Tissue	POO ^a^	POP ^b^	PPoO ^c^	POS	SOO	OOO	MOP ^d^	OOPo	PLO	PPP	SOS	PPS	POMa	SSS	Unknown ^e^
γ-hexalactone	0.20	−0.38	−0.31	0.11	0.31	0.24	−0.50	0.10	0.40	−0.22	0.26	−0.04	−0.11	−0.18	−0.25
γ-heptalactone	0.36	−0.30	−0.42	0.17	0.29	0.04	−0.39	−0.06	0.35	−0.01	0.24	−0.12	0.02	−0.11	−0.29
γ-octalactone	−0.30	0.09	0.16	−0.15	−0.30	−0.02	0.22	0.20	−0.05	0.12	−0.20	0.00	0.13	−0.30	0.31
γ-nonalactone	0.01	−0.02	0.20	−0.26	−0.18	0.08	0.11	0.17	0.21	−0.24	−0.30	−0.30	−0.41	−0.36	0.19
γ-decalactone	−0.26	0.23	0.42	−0.31	−0.43	−0.03	0.38	0.22	0.03	−0.19	−0.43	−0.20	−0.44	−0.37	0.41
δ-decalactone	0.06	0.28	0.00	0.12	−0.21	−0.32	0.21	−0.20	0.00	0.29	−0.09	0.08	0.14	−0.26	0.05
γ-undecalactone	0.21	0.29	−0.08	0.16	−0.13	−0.36	0.19	−0.25	−0.12	0.21	−0.01	0.13	0.03	0.01	−0.05
	**POO ^a^**	**POP ^b^**	**PPoO ^c^**	**POS**	**SOO**	**OOO**	**MOP ^d^**	**OOPo**	**PLO**	**PPP**	**SOS**	**PPS**	**POMa**	**SSS**	**Unknown ^e^**
C14:0	−0.62	0.77	0.52	−0.16	−0.77	−0.45	0.98	−0.10	−0.25	0.49	−0.51	0.28	0.25	−0.30	0.60
C14:1	−0.84	0.03	0.79	−0.74	−0.64	0.37	0.53	0.67	−0.12	−0.29	−0.73	−0.35	−0.39	−0.32	0.90
C15:0	−0.31	0.49	0.11	0.10	−0.59	−0.53	0.62	−0.30	−0.02	0.83	−0.26	0.39	0.79	−0.29	0.36
C16:0	−0.05	0.97	−0.06	0.47	−0.55	−0.90	0.71	−0.67	−0.23	0.74	0.00	0.76	0.37	−0.15	0.09
C16:1	−0.87	−0.11	0.92	−0.88	−0.61	0.51	0.49	0.80	−0.08	−0.33	−0.84	−0.53	−0.29	−0.36	0.89
C17:0	0.13	0.29	−0.36	0.44	−0.12	−0.55	0.18	−0.51	0.07	0.83	0.18	0.43	0.88	−0.02	−0.13
C18:0	0.66	0.15	−0.94	0.92	0.71	−0.47	−0.48	−0.76	−0.17	0.30	0.97	0.61	0.21	0.54	−0.83
C18:1	0.11	−0.88	0.12	−0.47	0.51	0.91	−0.69	0.65	−0.01	−0.84	−0.02	−0.73	−0.46	0.17	−0.11
C18:2	0.20	−0.52	0.10	−0.32	0.05	0.32	−0.34	0.34	0.93	−0.35	−0.22	−0.48	−0.22	−0.23	−0.08
C18:3	0.50	−0.58	−0.26	0.01	0.42	0.24	−0.57	0.02	0.57	−0.15	0.15	−0.27	0.16	−0.05	−0.42
Other	0.26	−0.40	−0.25	0.02	0.17	0.05	−0.32	0.01	0.69	0.14	0.05	−0.22	0.28	−0.20	−0.18
					**C14:0**	**C14:1**	**C15:0**	**C16:0**	**C16:1**	**C17:0**	**C18:0**	**C18:1**	**C18:2**	**C18:3**	**Other ^e^**
γ-hexalactone					−0.53	−0.22	−0.21	−0.34	−0.28	−0.10	0.21	0.22	0.54	0.41	0.31
γ-heptalactone					−0.39	−0.33	0.01	−0.22	−0.41	0.25	0.26	0.09	0.37	0.40	0.51
γ-octalactone					0.23	0.24	0.18	0.12	0.18	0.07	−0.21	−0.12	−0.03	−0.24	−0.01
γ-nonalactone					0.09	0.30	−0.08	0.03	0.11	−0.21	−0.25	0.02	0.15	−0.08	0.04
γ-decalactone					0.35	0.54	−0.09	0.24	0.36	−0.33	−0.37	−0.14	−0.07	−0.49	−0.24
δ-decalactone					0.13	−0.07	0.25	0.31	−0.07	0.20	0.03	−0.32	−0.08	−0.09	0.16
γ-undecalactone					0.20	−0.07	0.08	0.35	−0.16	0.18	0.07	−0.24	−0.24	−0.22	−0.03

**Table 4:** Details of beef samples used for testing. Beef samples were obtained from Type A and Type B Japanese Black cattle according to the aim of the study [11]. Type A Japanese Black cattle are a typical pedigree (non-Tajima) that exhibit excellent body weight growth. Type B is a closed breeding pedigree (Tajima) that is highly traded, and it has an excellent meat quality, that is, Kobe beef grade [40]. Meat quality grade is according to the carcass trading standards (The Japan Meat Rating Association, Tokyo, Japan). The grades range from 1 to 5, depending on marbling, meat color and brightness, meat hardness and texture, fat color, luster, and quality (higher values indicate high quality) [1].

**Analysis of Lactone, Fatty Acid, and TAG Composition in Marbled Area**					
**Group**	**Position**	**Meat Quality Grade**	**Number of Cattle**	**Slaughtered Age**	**Gender**
				**(Month)**	
Type A	Musculus longissimus	≥ A4	10	29.5 ± 0.9	Steer
Type B	Musculus longissimus	≥ A4	10	32.3 ± 1.3	Steer
**Analysis of LC-MS**					
**Group**	**Position**	**Meat Quality Grade**	**Number of Cattle**	**Slaughtered Age**	**Gender**
				**(Month)**	
Type A	Musculus longissimus	A3	3	28.2 ± 0.3	Steer
		A5	3	28.1 ± 0.5	Steer
	Adductor magnus	A3	3	28.2 ± 0.3	Steer
		A5	3	28.1 ± 0.5	Steer

## Data Availability

The data presented in this study are available in article and supplementary material.

## Supplementary Materials

The following are available online at https://www.mdpi.com/article/10.3390/metabo11040203/s1, Figure S1: Representative chromatogram of the GC-MS analysis, Figure S2: Representative chromatogram of the LC-MS and HPLC analyses.
