# Supplementary material for: Exploring the Lipids Involved in the Formation of Characteristic Lactones in Japanese Black Cattle
Source: Metabolites. 2021 Mar 29;11(4):203. doi: 10.3390/metabo11040203 (PMC8067244; doi:10.3390/metabo11040203)
Supplement: Supplementary file 1 [file metabolites-11-00203-s001.pdf]

Supplementary data

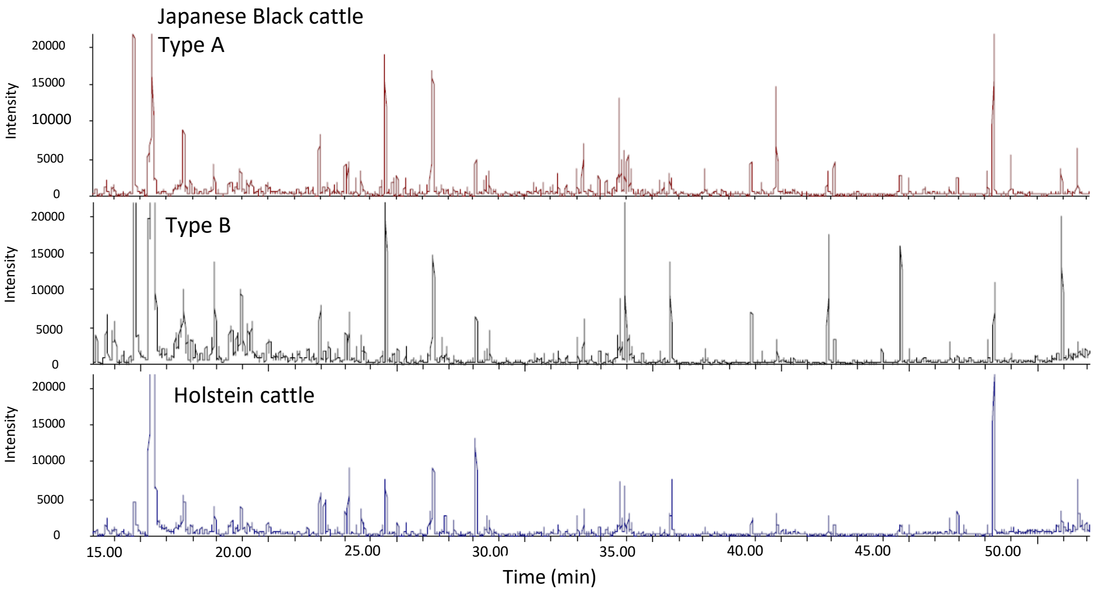

Figure S1. Representative chromatogram of the GC-MS analysis.

The chromatogram shows the total ions in Type A and Type B Japanese Black cattle and Holstein cattle. The number and intensity of chromatographic peaks indicate the difference in aroma components between fat tissues.

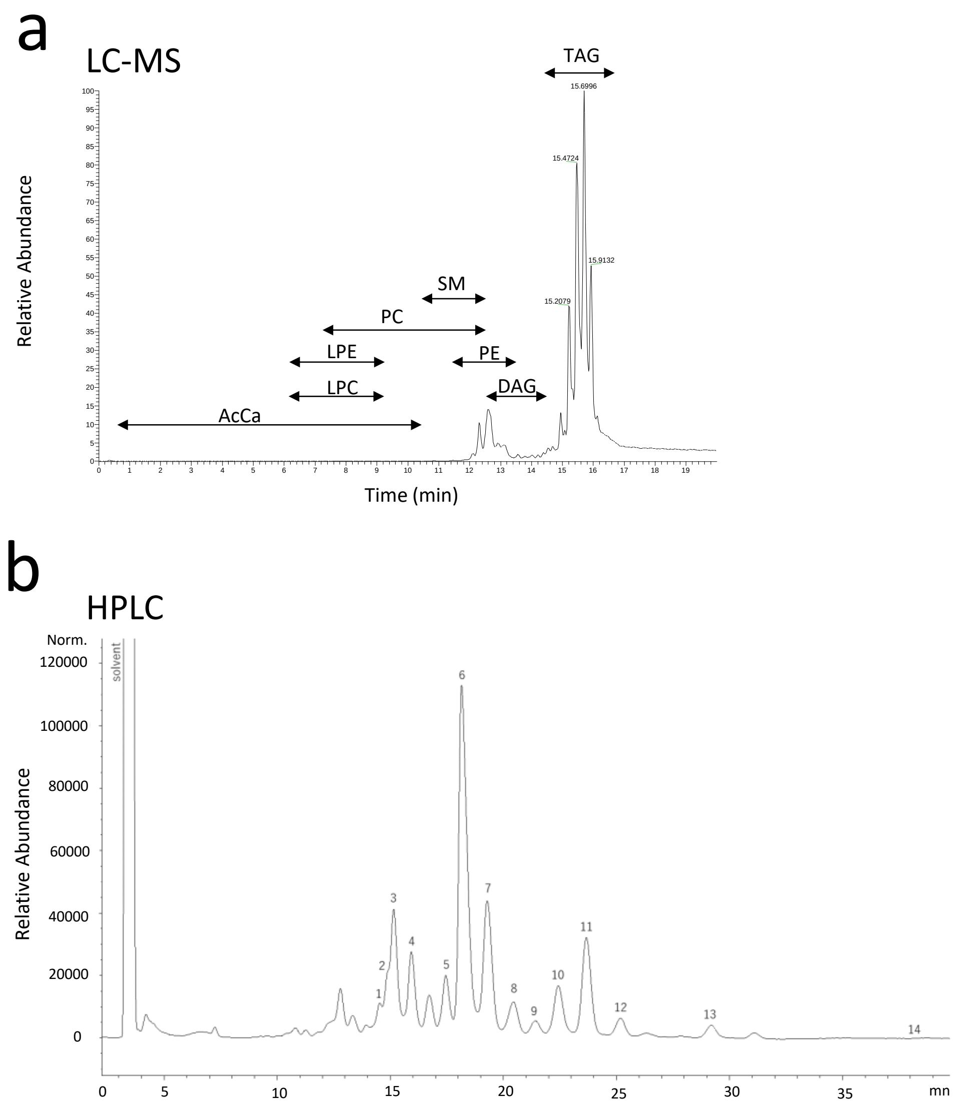

Figure S2. Representative chromatogram of lipids determined using the LC-MS and HPLC analysis.
a. Chromatogram of the total ions of lipids identified by LC-MS. Double-headed arrows indicate the detected range of peaks for each lipid class. acylcarnitine (AcCa), lysophosphatidylcholine (LPC), lysophosphatidylethanolamine (LPE), phosphatidylcholine (PC), phosphatidylethanolamine (PE), sphingomyelin (SM), diacylglyceride (DAG), triacylglyceride (TAG). b. Chromatogram of the TAG molecular species identified by HPLC. The number of elution peaks indicates the TAG molecular species. 1. OOPo, 2. PLO, 3. PPoO + MOO, 4. MOP + PLP, 5. OOO, 6. POO + SLO, 7. POP + PLS, 8. PPP, 9. POMa, 10. SOO, 11. POS, 12. PPS, 13. SOS, and 14. SSS. The TAGs in parentheses indicate the TAG molecular species that are expected to be slightly mixed owing to their retention times. oleic acid (O), palmitic acid (P), stearic acid (S), palmitoleic acid (Po), linoleic acid (L).
